# Supplementary material for: Are carbon emissions trading and green financial instruments synergistic? -Comprehensive quantitative research based on content analysis
Source: PLoS One. 2024 Mar 7;19(3):e0298601. doi: 10.1371/journal.pone.0298601 (PMC10919601; doi:10.1371/journal.pone.0298601)
Supplement: S1 Data — (ZIP) [file pone.0298601.s001.zip › Data/地级市Z_F工作报告.zip!/║╙─╧/╫ñ┬φ╡Ω╩╨/└┤╫╘╣1⁄2╓┌║┼í╛┬φ┐╦╩2╛▌═°í┐_2013.docx]

各位代表：

　　现在，我代表市人民政府，向大会作工作报告，请予审议，并请各位政协委员提出意见。

　　一、2012年政府工作回顾

　　过去的一年，面对复杂严峻的经济形势，在省委、省政府和市委的正确领导下，全市上下坚持以科学发展观为指导，认真贯彻落实国家、省一系列政策措施，按照“三个一”的工作思路，把保增长放在各项工作的重中之重，强力推进“十项重点工作”，建立健全“四项保障机制”，齐心协力，攻坚克难，扎实工作，较好地完成了市三届人大一次会议确定的目标任务。全年完成生产总值1387亿元，增长10.4%，其中一、二、三产业增加值分别增长4.6%、14.7%和9%；地方财政总收入97.2亿元，增长24.7%，地方公共财政预算收入58.9亿元，增长25.1%；完成固定资产投资817.6亿元，增长23.4%；社会消费品零售总额514.24亿元，增长15.9%；城镇居民人均可支配收入17671元、农民人均纯收入6599元，分别增长11.9%和13.7%。

　　(一)项目建设成效显著。坚持把项目建设作为总抓手，深入开展“项目建设推进年”活动，集中开工了五批164个投资亿元以上重点项目，先后召开39次周例会，解决了200多个影响项目建设的突出问题。全市171个重点项目完成投资274.2亿元，是上年的2.37倍，104个亿元以上工业重点项目完成投资186.9亿元，是上年的2.63倍，实现了年初确定的“两个翻番”目标。30多个亿元以上重点工业项目竣工投运，天方药业新型原料药、华润风电、鼎瑞模塑等续建项目快速推进，银泰电动汽车、保税中心、海川玻璃等重大项目开工建设。石武高铁驻马店西站正式运营，京港澳高速至上蔡、正阳连接线建成通车，实现了所有县城20分钟上高速目标。

　　(二)工业发展态势良好。制定出台了一系列应对措施，强化企业服务，搞好要素保障，帮助解决问题，同时引导企业加强管理、挖潜增效、逆势而上、强渡难关，促进了工业的快速发展。全市完成规模以上工业增加值415.5亿元，增长18.6%，增速居全省第二位；实现主营业务收入1671.8亿元、利润121.7亿元，分别增长20.1%和14.9%。积极推进工业转型升级，大力培育支柱产业和新兴产业，食品、医药、能源和煤化工、装备制造、建材、轻纺六大支柱产业增加值的比重在50%以上。实施了一批新兴产业项目，企业自主创新能力持续提升。产业集聚区主导产业逐步显现，支撑带动能力明显增强，完成规模以上工业增加值225亿元，固定资产投资520亿元，已成为工业经济发展的重要增长极。

　　(三)农村经济全面发展。认真落实强农惠农富农政策，大力推进现代农业发展，粮食生产实现九连增，总产达700.29万吨，增长2.25%，肉、蛋、奶产量分别增长1.6%、1%和6%。现代农业产业化集群培育工程全面启动，农业产业化组织达到1738家，市级以上农业产业化龙头企业316家，其中国家级2家、省级44家。完成造林15.4万亩，林业产值26.2亿元。新增有效灌溉面积87.6万亩，新增旱保田61.6万亩，打井和配套机井14671眼，治理水土流失面积85平方公里。改造建设农村公路390公里，新发展农村沼气近1万户，解决了农村50多万人口安全饮水问题，扶贫开发和移民后期扶持工作取得新成效。

　　(四)新型城镇化步伐加快。坚持以新型城镇化为引领，加强规划、建设与管理，促进了城乡互动、良性发展，全市城镇化率达到33.4%，较上年提高1.9个百分点。按照“一中心五组团”和建设山水园林、生态宜居城市的要求，认真做好各项规划编修，中心城区总规修编通过专家评审，新区建设方案已经省委、省政府批准，完成了商务中心区等专项规划编制工作。中心城区七大功能片区整体开发全面启动，实施了一批市政道路、园林绿化等基础设施项目，全年完成投资33.9亿元，增长78.4%，建成区面积接近70平方公里。中心城区管理体制逐步理顺，“三项整治”、“三乱两超”综合治理、县乡环境卫生集中整治取得阶段性成效。市、县污水处理厂实现达标排放，城镇污水和生活垃圾无害化处理率均在85%以上。县城和小城镇建设步伐加快，城镇功能逐步完善，对人口和产业发展的承载力明显提升。新型农村社区建设稳步推进，全市新开工建设新型农村社区51个，其中万人社区21个、5000人社区30个。

　　(五)改革开放深入推进。国有企业战略重组不断深化，非公有制经济快速发展，行政管理体制改革扎实推进，事业单位改革有序展开，医药卫生及文化体制改革迈出新步伐。积极创新投融资、土地利用、人力资源保障机制，全年新增贷款103.7亿元，增长19.2%，利用资本市场融资50.5亿元，13亿元城投债顺利发行；土地整治、集约节约用地水平不断提高，建设用地需求得到较好保障；完成职业技能培训19.8万人，新增农村劳动力转移就业7万人。深入开展大招商活动，新签约亿元以上招商项目236个，总投资1340.5亿元，签约项目数量、投资总额均实现翻番；累计到位省外资金158亿元，增长23.8%。全国“农洽会”取得圆满成功，我市签约亿元以上重点项目97个，总投资526亿元。全年实际利用外资2.7亿美元，增长22.3%；完成出口2.16亿美元，增长15.5%。

　　(六)民生得到持续改善。进一步加大民生投入，十项重点民生工程全面落实，全市民生支出206.7亿元，增长30.4%，占公共财政预算支出的76.9%。社会事业全面发展，全年组织实施各类科技计划项目182项，研究开发新技术、新工艺131项。中小学、幼儿园规划建设、薄弱学校改造和职教园区建设加快推进，广电中心、中心医院新病房楼基本建成，市群艺馆、博物馆投入使用。就业和社会保障得到加强，全市城镇新增就业8.1万人，失业人员再就业2.6万人，城镇登记失业率3.16%；城乡居民社会养老保险实现全覆盖，城乡低保标准进一步提高，新农合参合率达到98.16%；新开工保障性住房2.48万套，已建成1.1万套。人口自然增长率控制在6.5‰以内，继续稳定了低生育水平。交通事业快速发展，完成投资27.19亿元，其中高速公路6.4亿元，干线公路13.45亿元。广播电视、新闻出版、文学艺术、体育事业健康发展。加强和创新社会管理，注重畅通信访渠道，积极化解各类矛盾纠纷，认真落实安全生产各项措施，不断强化食品药品监管，扎实推进平安建设，严厉打击各类违法犯罪活动，保持了社会大局的和谐稳定。

　　加强民主法制建设，自觉接受人大监督、政协民主监督和社会监督，认真办理人大代表建议和政协提案。加强精神文明建设，精神文明创建活动广泛开展。加强廉政建设，政风行风明显好转。高度重视驻驻部队和国防后备力量建设，深入开展拥军优属、拥政爱民和军民共建活动，密切了军政军民关系，再次荣获“全国双拥模范城”称号。民族宗教、旅游、审计、统计、气象、史志、档案、侨务、人防、红十字会、妇女儿童、残疾人等各项工作，都取得了新的成绩。

　　各位代表！回顾一年来的工作，成绩来之不易。一年来，面对国际金融危机持续蔓延、市场需求持续下降，特别是我市结构性矛盾凸显、资源环境约束加剧、区域竞争日趋激烈、社会管理难题增多的严峻形势，全市上下坚持以科学发展观统领发展全局，把保增长作为各项工作的重中之重，科学谋划、积极应对，综合施策、统筹推进，在加快崛起、赶超发展中进行了不懈探索，付出了艰辛努力，经济社会发展保持了好的趋势、态势和气势。这是省委、省政府和市委正确领导的结果，是市人大、市政协积极支持的结果，是全市广大干部群众奋力拼搏的结果。在此，我代表市人民政府，向工作在各条战线上的广大工人、农民、知识分子、干部、驻驻部队指战员、武警官兵、公安民警，向所有关心支持政府工作的各位人大代表、政协委员、离退休干部和民主党派、工商联和无党派人士，表示崇高的敬意和衷心的感谢！

　　各位代表！在肯定成绩的同时，我们也清醒地看到，当前我市人口多、基础差、底子薄和农业大市、工业小市、财政穷市的基本市情尚未根本改变，经济社会发展仍面临不少困难和问题。主要表现在：经济总量小，人均水平低，工业化、城镇化支撑引领作用不强，加快发展的任务十分繁重；产业层次较低，发展方式粗放，创新能力不足，质量效益不高，结构调整的任务非常艰巨；资源、环境、要素瓶颈制约日益突出，推动科学发展的体制机制亟待完善；社会事业欠账较多，一些涉及群众切身利益的就业、就学、就医、住房等问题尚未得到很好解决，民生改善任重道远；一些政府工作人员的能力素质、工作作风、工作方法与新形势新任务的要求不相适应，一些部门服务意识较差、办事效率不高，少数干部奢侈浪费、消极腐败现象依然存在。对此，我们一定高度重视，采取有效措施认真加以解决，努力开创各项工作的新局面。

　　二、2013年的目标任务和重点工作

　　2013年是全面贯彻落实十八大精神和实施《中原经济区规划》的开局之年，也是为全面建成小康社会奠定基础的重要一年。作为发展中的人口大市，既要保持较快的经济增长速度，努力扩充经济总量，逐步缩小与全省、全国的差距，又要切实转变发展方式，持续推进结构调整和产业升级，不断提高发展的质量和效益。尽管面临的困难和挑战前所未有，但我市已具备实现赶超发展的良好基础，今后一个时期仍处于重要战略机遇期。只要我们抢抓机遇、开拓进取、扎实工作，就一定能够夺取科学发展、务实发展、赶超发展的新胜利！

　　今年政府工作的指导思想是：深入贯彻落实党的十八大和中央、省委、市委经济工作会议精神，坚持以邓小平理论、“三个代表”重要思想和科学发展观为指导，以中原经济区建设为契机，以提高经济增长质量和效益为中心，按照“四个坚持”的发展思路和“持续求进、着力提升”的总体要求，紧紧围绕主题主线，着力招商引资、着力项目建设、着力调整结构、着力改革创新、着力改善民生，在加快信息化进程中持续推进“三化”协调发展，为实现富民强市加快崛起、全面建成小康社会奠定坚实基础。

　　今年经济社会发展的主要目标是：生产总值增长10%左右，规模以上工业增加值增长17%左右，全社会固定资产投资增长21%，地方财政公共预算收入增长13%，社会消费品零售总额增长15%，城乡居民收入增长9%以上，城镇新增就业6万人以上，人口自然增长率控制在6.5‰以内，居民消费价格涨幅控制在3.5%以内，城镇化率提高1.5个百分点以上，节能减排完成省定目标。

　　按照上述指导思想和目标任务，做好今年的政府工作，必须坚持总思路、把握总要求、突出八重点。坚持总思路，就是“四个坚持”的发展思路，即坚持富民强市、加快崛起的发展战略，坚持“三化”协调、统筹推进的发展路径，坚持开放招商、项目带动的发展举措，坚持以人为本、求真务实的发展要求。把握总要求，就是持续求进、着力提升的总体要求，即持续当前好的精神状态、好的发展势头、好的发展氛围，在加快发展、做大经济总量的同时，更加重视转型升级、提高质量效益，着力在加快发展上提升、在优化结构上提升、在统筹推进上提升、在改善民生上提升，努力实现经济社会持续较快健康发展。突出八重点，就是着力抓好以下八项重点工作：

　　(一)承接产业转移，大力实施开放招商。坚持把开放招商作为“一举求多效”的重要手段，始终放在经济工作的首位，不断创新招商理念，转变招商方式，推动开放招商取得新成效。更加注重招商的针对性。积极适应形势变化，采取“走出去、请进来”相结合，以港澳台、珠三角、长三角、京津冀为区域招商重点，瞄准中外500强、央企和行业领军企业，围绕主导产业延链补链，集中力量引进一批在产业链中起关键作用的重大项目。更加注重招商质量。加快从招商向选商转变，突出主导产业招商、战略性新兴产业招商和招大引强，着力引进一批竞争力强、成长性好、产业关联度高的项目，力争在引进50亿元以上的龙头型、基地型项目上实现突破。围绕办好全国“农洽会”，有针对性地组织好市县两级招商活动，宣传推介一批重大项目，引进落户一批知名企业，提升办会层次，扩大会议影响。更加注重招商实效。进一步完善招商机制，改进招商措施，大力开展专业化、集群式招商。对一些重大项目、重点产业，主要领导挂帅，组成专业队伍进行一对一招商。强化招商引资责任，继续实行县区主要领导“二分之一”工作法，确保招商引资工作取得更大成效。更加注重多领域对外开放。坚持引资、引智、引技相结合，鼓励引导社会资本进入社会事业领域，实施科技开放合作工程、社会资本办学办医行动计划，吸引国内外高校、科研单位在我市设立研发中心，推动一批医疗、教育项目落地。支持天方药业、华中正大、中集华骏、康茂工艺等企业扩大出口规模，积极培育瑞升家俬、宝兰德皮具、联播生物制品等新的出口增长点，加快公共保税中心建设，促进外经外贸健康发展。

　　(二)着力扩大投资，强力推进项目建设。把扩大投资作为拉动经济增长的重要举措，以项目建设为总抓手，继续扩大投资规模，优化投资结构，增强发展后劲。今年初步安排重点建设项目239个，总投资1742亿元，年度计划投资529亿元，其中亿元以上工业项目132个，总投资796亿元，年度计划投资303亿元。通过实施项目建设，努力实现“两个突破”，即全市固定资产投资突破1000亿元、全市产业集聚区固定资产投资突破700亿元。工业方面，抓好天方药业抗生素原料药、昊华骏化年产30万吨合成氨技改、电动汽车产业园、富兴电子、施科特光电、恒都食品、立马电动车等续建项目建设，确保完成年度建设任务；加快鼎瑞模塑、联顺食品、后羿制药等计划竣工项目建设进度，确保按期竣工投产；开工建设昊华骏化醋酸制乙醇、华润集团液化天然气、天方药业多功能中试车间、燕京啤酒二期等项目。水利方面，加快洪汝河等骨干河道治理及老王坡、杨庄、蛟停湖三大滞洪区建设，抓好病险水库、水闸除险加固和重点中小型河道、重点平原洼地治理，推进大型灌区续建配套和节水改造项目建设。交通方面，加快京港澳高速驻马店段扩建和驻马店出入口北迁工程建设，做好周驻南和息邢高速驻马店段、107国道升级改建等项目前期工作，推进干线公路改造升级、运输场站和农村公路建设。能源方面，抓好安里煤矿、吴桂桥煤矿洗煤厂、华润新能源泌阳风电、国能集团上蔡30万千瓦生物质发电等项目建设。社会事业方面，抓好市职教园区、体育中心、报业大厦、第二中医院、妇幼保健中心和市殡仪馆等项目建设。同时围绕中原经济区建设，依托我市资源、区位等优势，继续挖掘、筛选、包装一批重大项目，力争有更多项目进入国家、省扶持计划。认真落实领导分包重点项目、联审联批和周例会制度，优化建设环境，提高办事效率，促进项目落地。强化项目管理，把除房地产以外的所有亿元以上项目全部纳入重点项目管理，继续开展项目集中开工和项目观摩点评活动，进一步激发各地抓项目建设的积极性。

　　(三)统筹城乡发展，加快新型城镇化进程。按照“一中心五组团”战略布局，以中心城区建设为龙头，推动县城、重点镇和新型农村社区协调发展。加快中心城区发展。尽快完成中心城市总体规划报批，高标准完成新区规划编制，继续抓好专项规划及重点地段控制性、修建性详规编制，提高控规覆盖率。加大招商融资力度，充分发挥城投债效益，加快城市新区和中心城区七大功能片区开发建设，实施好城市建设十大重点工程和中心城区六件惠民实事，力争中心城区基础设施建设投资完成60亿元以上。以铜山大道、驿城大道、纬十路、商桐路等快速通道建设为重点，打造30分钟交通圈，加快“一中心五组团”格局的形成。加强城市游园、绿地和生态水系建设，实施练江河、小清河、冷水河综合治理，改善生态环境，打造山水园林、生态宜居城市。大力实施市政道路、环卫设施建设工程，积极推进薄山水库引水及第三水厂、第三污水处理厂等重点项目建设。建立健全城市管理长效机制，深入推进城市精细化管理，进一步改善城市面貌，争创省级文明城市。加快县城和重点镇建设。实施城镇基础设施扩容提升工程，拉大城市框架，形成产业集聚区、县城新区和旧城区“三位一体”发展格局。统筹规划一批功能片区，搞好整体开发建设，注重地域、文化特色，打造个性化城市。加快重点镇道路、水电等基础设施建设，完善城镇功能，强化产业支撑，增强吸纳集聚能力。积极稳妥推进新型农村社区建设。按照“产业为基、就业为本、生计为先”原则，坚持分类指导、规划先行、政策引领、示范带动，继续以产业集聚区、城乡接合部和旅游景区周边区域为重点，积极探索重点镇社区建设模式，确保新型农村社区建设健康发展。有序推进农村人口转移。鼓励引导农业人口就近城镇化，强化产业支撑，推动以产兴城，更好地解决进城农民的就业问题；探索建立有利于农村人口有序转移的体制机制，突出住房牵动和学校牵动，把具备条件的农业转移人口纳入城镇住房保障体系，加强城镇学校、医院等公共服务设施建设，切实解决好转移人员的安居、子女就学、社会保障等问题。

　　(四)调整优化结构，强化新型工业化主导作用。以工业转型升级为重点，以产业集聚区建设为载体，以提高运行质量和效益为目标，着力提高工业整体竞争力。加快工业转型升级。制定实施工业转型升级行动计划，坚持以技术创新、管理创新、制度创新改造提升传统产业，加快培育电子信息、生物医药、新材料、新能源等新兴产业，不断提高高新技术和高附加值产品比重。从今年开始，市县两级财政每年安排5000万元资金，重点奖励企业和科研单位各种创新、研发以及有重大突破的技术攻关，培养、改造、孵化我市的高新技术产业。积极引导资金、技术、人才等要素资源向优势产业集中，培育壮大农副产品加工、能源化工、医药、建材、轻纺、装备制造等优势产业。继续深化重点企业合资合作，加大项目谋划建设力度，做大做强骨干企业，确保主营业务收入超百亿元企业1家、超50亿元企业3家、超10亿元企业20家。加强产业发展载体建设。继续按照“四集一转”要求，突出龙头带动、链式发展、完善配套、强化保障，大力实施产业集群培育工程，围绕主导产业培育和延链补链，认真落实项目统筹布局及“退二进三”政策，加强基础设施和公共服务平台建设，力争全市产业集聚区主营业务收入达到1400亿元，有5个产业集聚区主营业务收入超过100亿元。推进自主创新能力建设。积极培育创新型企业，支持中集华骏、昊华骏化、平煤蓝天、白云纸业等争创国家级企业技术研究中心，华顺阳光、华中正大等争创省级工程技术研究中心，建成昊华骏化博士后工作站。持续推进品牌带动战略，加强质量提升和标准化建设，加快实施一批高技术专项、工业技改和科技创新项目。搞好经济运行调节。推动企业服务常态化、制度化、长效化，积极帮助企业解决实际困难。加强企业产销对接，引导上下游企业建立战略联盟。做好煤电油气运产需衔接，保障生产要素和物资供应。规范各类检查、评比、收费行为，切实减轻企业负担。积极推进信息化建设。加强信息化和工业化融合示范工程建设，创建一批示范企业，用信息化促进产业优化升级。加快推进数字城市地理空间框架建设，力争年内建成数字化城市管理系统。

　　(五)强化农业基础，提升现代农业发展水平。以建设国家级现代农业示范区为主线，突出重点，打造亮点，加快现代农业发展，促进农业大市向农业强市转变。巩固提高粮食综合生产能力。坚持以高标准粮田“百千万”建设工程为载体，加快实施国家新增千亿斤粮食生产能力规划、中低产田改造、小农水建设和节水农业工程，建设40个小麦、40个玉米万亩高产创建示范区，新增高标准粮田100万亩、有效灌溉面积70万亩、旱保田55万亩。认真落实农业生产扶持政策，积极推进适度规模经营，稳定提高粮食综合生产能力，确保粮食总产稳定在140亿斤以上。加快发展现代农业产业集群。以优质粮油、蔬菜、食用菌、夏南牛、优质生猪、烟叶、茶叶等为重点，大力实施现代农业产业化集群培育工程，通过龙头带动、专业合作、协作配套、精深加工、打造品牌，着力建设一批规模化、标准化、专业化农产品加工生产基地。大力扶持农业产业化龙头企业发展，进一步壮大农畜产品加工业，力争培育农业产业集群20个以上，提高农业综合效益。综合运用政策引导、项目扶持、奖励补助等手段，扶持联户经营、专业大户、家庭农场。按照积极发展、逐步规范、强化扶持、提升素质的要求，加快发展新型农民合作组织，构建集约化、专业化、组织化、社会化相结合的新型农业经营体系。加快建设现代农业服务体系。加强农业科技、质量安全、市场信息等公共服务体系建设，积极发展农业保险，扩大基层农技推广、动植物防疫和农产品质量安全检测体系覆盖面，加快推进主要农作物生产全程机械化。继续改善农村生产生活条件。加强农村安全饮水、道路、供电、通信、沼气等基础设施建设，力争解决49.5万人的安全饮水问题，新增农村沼气用户1万户，解决脱贫人口13.87万人。

　　(六)加快服务业发展，培育新的经济增长点。把服务业发展放到更加重要的位置，进一步突出特色、整合资源、拓展领域、打造品牌，促进服务业加快发展。加快商务中心区和特色商业区建设。积极推进商贸流通、餐饮、住宿等传统服务业升级改造，加快启动商务中心区和特色商业区开发建设，重点围绕服务特色商品交易和主导产业发展，尽快实施一批建设项目，培育一批现代服务业产业集群。加快发展旅游产业。完善并组织实施《全市旅游业发展总体规划》，全面启动大嵖岈山旅游产业集聚区、西部山区休闲度假旅游、宿鸭湖湿地生态旅游和西南部山区乡村旅游产业带开发建设，搞好项目包装，提升文化内涵，打造旅游精品，拓宽旅游市场，努力建设山水生态、休闲驿站的美丽天中。积极发展金融业。继续做好银企对接，切实加大信贷投放。积极引进国内金融机构，争取在引进股份制银行方面实现新突破。支持国有商业银行完善网点布局，实施驻马店银行创优升级工程，抓好农村信用社改革，推进农村商业银行组建工作，力争实现村镇银行全覆盖。加强政府投融资平台建设，增强融资能力。加大企业上市培育力度，力争蓝天燃气上市。大力发展保险事业，充分发挥经济“助推器”和社会“稳定器”作用。加快推进物流业发展。做好物流业规划编制，完善与海关协作机制，加快保税中心建设，打造豫南区域性物流中心。加强农产品流通体系建设，大力实施“万村千乡”市场工程、“新网工程”、“双进工程”，积极开展农超对接、农社对接，加快建设一批大型农产品批发市场和各类专业批发市场，培育一批大型涉农商贸企业。

　　(七)持续改善民生，维护社会和谐稳定。强化民生改善和社会管理创新，继续抓好“十项重点民生工程”，解决好人民群众最关心最直接最现实的利益问题。加强就业和社会保障工作。实施积极的就业政策，多渠道增加就业岗位，确保全年城镇新增就业6万人，下岗失业人员再就业1.8万人，新增农村劳动力转移就业7万人。全面实现失业保险市级统筹，完善医疗保险市级统筹，积极推进全市就医一卡通。巩固城乡居民社会养老保险成果，加强社会保险基金征缴，认真落实各项社会保险待遇。继续提高城乡低保、农村五保供养和优抚对象待遇，不断完善社会救助体系，切实保障困难群众的基本生活。积极推进城区旧城改造、城中村改造和棚户区改造，持续抓好以公租房为重点的保障房建设，全年建设保障性住房项目33个24393套，不断改善群众居住条件。千方百计增加居民收入。稳步推进收入分配制度改革，完善机关事业单位工资收入分配制度，健全职工工资正常增长和支付保障机制，提高低收入职工、基层一线职工工资水平。持续推进农业劳动力向非农产业转移，增加工资性收入；全面落实涉农补贴，降低农业成本，提高农业生产效益，增加农民经营性收入；完善政策机制，提高农民在土地增值收益中的分配比例；增加城乡居民财产性收入，不断提高居民消费能力。大力发展各项社会事业。深入实施学前教育三年行动计划，优化农村中小学布局，加快推进城乡义务教育扩容改造工程，促进新一轮义务教育均衡发展。继续实施普通高中改造工程，改善薄弱高中办学条件。加强职业教育和职教园区建设，完成驻马店高级技工学校搬迁，基本完成汝南幼儿师范、驻马店卫校、驻马店农校一期工程，开工建设职业技术学院、驻马店财经学校，切实做好驻马店幼儿师专和驻马店技师学院申办工作。积极推进医疗卫生事业“三年九大攻坚行动计划”，继续实施一批公共卫生、疾病防控和县、乡镇医院改扩建、村卫生室标准化建设项目，切实做好基层卫生人才培养工作，提高城乡医疗卫生服务水平。完善政策机制，加强养老托老设施建设，促进养老事业发展。开工建设市残疾人康复中心，落实好0~6岁残疾儿童抢救性康复任务。稳步推进公益性文化事业单位改革，抓好城乡文化基础设施项目建设，大力发展文化事业和文化产业。广泛开展全民健身活动，促进群众体育和竞技体育全面发展。认真落实计划生育政策，稳定低生育水平，实现人口长期均衡发展。加强生态文明建设。把生态文明建设放在更加突出的位置，全面落实节约资源和保护环境基本国策，推进绿色、循环、低碳、可持续发展，为人民群众提供宜居舒适生活环境。做好生态市规划编制工作，继续抓好“三乱两超”整治、西部山区生态修复及县、乡、村环境综合治理，积极创建生态县、生态乡、生态村。强化节能减排和环境保护，加强环境自动监控系统和监测网络建设，全面推进重点领域节能降耗，加快淘汰落后产能，严格环保准入和污染物排放标准，加强水源地保护，完成9个县区污水处理厂升级改造。推进天然林保护、退耕还林、水土保持等项目建设，完成造林18.5万亩，森林抚育8.4万亩，建设林业生态市。特别是经历了一个时期的雾霾天气之后，我们切身感到，加强环境保护，建设生态文明，不仅涉及经济社会发展，而且已成为各级政府的重大政治责任。我们一定要下大决心，采取坚决措施，认真做好工作，在保持经济较快增长的同时，使我们驻马店的水更清、山更绿、天更蓝，坚决不干吃祖宗饭、断子孙路的事。加强和创新社会管理。深入研究新形势下的社会管理规律，不断提高社会管理科学化水平。增强城乡社区服务功能，完善村(居)民自治制度，强化企事业单位、社会团体的社会管理服务职责，夯实基层社会管理基础。切实加强精神文明建设，广泛开展理想信念和爱国主义、集体主义、社会主义教育，深化群众性精神文明创建活动，不断提高全民文明素质。畅通群众诉求表达、利益协调、权益保障渠道，完善信访机制，做好信访工作。加强应急管理，提高突发事件应对处置能力。加强安全生产和食品药品安全监管，坚决遏制重特大事故发生。深入开展平安驻马店建设，强化社会治安综合治理，依法打击违法犯罪活动，持续提升人民群众的安全感和满意度，确保社会大局和谐稳定。

　　(八)推进改革创新，增强科学发展的动力。把深化改革作为加快转变经济发展方式的关键，突出重点领域和关键环节改革创新，着力构建系统完备、科学规范、运行有效的体制机制。进一步深化各项改革。认真做好国有企业脱钩改制，支持、引导非公有制经济健康发展。深入推进医药卫生体制改革，以健全医保、规范医药、创新医疗为重点，提高城乡居民医保补助和报销标准，开展城乡居民大病保险试点，巩固完善基本药物制度和运行新机制，全面启动县级公立医院综合改革。推进农业生产经营组织创新，制定支持土地流转、规模经营的政策措施，促使农村土地向龙头企业、农民专业合作社和种植、养殖大户集中。积极推进财税、运输、价格、收入分配等领域的改革。持续创新要素保障机制。完善融资机制，努力扩大信贷投放和融资规模，进一步拓宽资本市场直接融资渠道，保障项目建设和企业发展资金需求。完善土地保障机制，加强土地计划调控，优先支持重点项目、重点企业和发展载体建设用地，积极稳妥推进人地挂钩试点，扎实开展农村土地整治，严格执行各类工程项目建设用地标准，提高土地利用效率。完善人力资源开发机制，加快实施职教攻坚和全民技能振兴工程，加大技能培训力度，不断提高劳动者素质。

　　继续加强国防教育和国防后备力量建设，支持驻驻解放军、武警部队、预备役部队、民兵和人民防空建设，深入开展双拥共建，巩固军政军民团结，促进军民融合式发展。支持工会、共青团、妇联等人民团体发挥桥梁纽带作用，注重发挥工商联等社团作用。继续做好人事、审计、统计、民族宗教、外事侨务、红十字会、档案、史志、地震、气象等工作。

　　三、加强政府自身建设，不断提高施政水平

　　把改进工作作风、提升能力水平作为加强政府自身建设的关键，认真落实中央、省委和市委关于改进工作作风、密切联系群众有关规定，坚持用领导方式转变加快发展方式转变，落实“三具两基一抓手”工作方法，深入推进“两转两提”，全面提升政府工作水平。

　　(一)继续转变政府职能。更加重视社会管理和公共服务，正确处理政府和市场的关系，减少对微观经济活动的干预，推动政府职能向创造良好发展环境、提供优质公共服务、维护社会公平正义转变，推进服务型政府建设。健全部门职责体系，严格定机构、定职责、定编制，强化部门职能，提升服务水平。继续深化行政审批制度改革，进一步清理审批事项，简化审批程序，压缩办事时限，提高办事效率。加强政务服务体系建设，深化政务公开，为人民群众和市场主体提供优质便捷高效服务。

　　(二)不断强化依法行政。落实依法治国基本方略，严格在宪法和法律范围内活动，依照法定权限和程序行使权力、履行职责、规范行为，自觉用法治思维和方式调节经济运行、管理社会事务。严格规范、公正、文明执法，充分发挥行政监察和审计监督的作用，认真落实执法责任制和纠错制度，推行服务型执法。做好行政调解等工作，办好行政复议案件。深入开展法制宣传教育和公务员培训，建设高素质的公务员队伍，不断提升服务质量和公务员的依法行政能力。

　　(三)持续提升行政效能。积极应对形势变化，主动站位全局，加强调查研究，注重战略谋划，健全决策机制和程序，推进科学决策、民主决策。抓住主要矛盾，区分轻重缓急，合理配置资源，调动各方力量，创造性开展工作，不断提高有效运作能力。进一步完善责任目标考核体系，强化政府绩效管理，继续实施项目建设、城市建设和县域经济发展“一票否决”制度，加大督查督办和奖惩力度，对务实重干、成绩突出的表彰奖励，对失职渎职的追究责任。严肃查处破坏发展环境的行为，依法打击各种黑恶势力，重点整治行政不作为、乱作为和执法犯法、吃拿卡要等问题，进一步优化发展环境，促进经济社会发展。

　　(四)大力改进工作作风。积极开展为民务实清廉教育实践活动，树立政府良好形象。坚持求真务实，说实话、办实事、求实效，坚决杜绝弄虚作假、做表面文章、搞形式主义。坚持密切联系群众，问政于民、问需于民、问计于民，办好顺民意、解民忧、惠民生的实事。改进调研方式，轻车简从、简化接待，多到情况复杂、矛盾集中、群众意见多的地方帮助解决问题。改进文风会风，减少检查评比，真正把精力用在谋大事、抓落实、搞服务、促发展上，努力创造出经得起历史和人民检验的业绩。厉行勤俭节约，反对铺张浪费，严格控制行政经费等一般性开支，严格控制楼堂馆所建设，严格执行领导干部住房、车辆配备等有关规定。认真落实党风廉政建设责任制，完善惩防体系，加强制约监督，规范权力运行，防控腐败风险，坚决惩处权钱交易等违法违规行为，努力做到干部清正、政府清廉、政治清明。

　　各位代表！作为人民的政府，我们一定严格执行市人大及其常委会的决议决定，自觉接受法律和工作监督，主动接受市政协的民主监督，虚心接受人民群众社会监督和媒体舆论监督，认真办理人大代表建议和政协提案，广泛听取各民主党派、工商联、无党派人士、人民团体的意见，让人民更有效监督政府，使政府更好服务人民，决不允许慵懒散奢，决不允许弄虚作假，决不允许损害群众利益！

　　各位代表！党的十八大指引我们踏上新的历史征程，全面建成小康社会目标催人奋进。面对驻马店890万人民的新期待，我们深感责任重大，使命光荣。让我们在省委、省政府和市委的正确领导下，进一步解放思想、开拓创新，凝聚力量、扎实工作，努力创造经得起实践和历史检验的业绩，努力使驻马店人民过上更加美好的生活，不断开创驻马店富民强市、加快崛起的新局面！
